# Antimicrobial resistance patterns and risk factors of *Enterococcus faecium* bloodstream infections in hospitalized patients in a Chinese tertiary care hospital

**DOI:** 10.3389/fpubh.2026.1771155

**Published:** 2026-05-05

**Authors:** Chuyu Lao, Zihuan Li, Guanwen Lin, Cuiqiong Fan, Maorui Lin

**Affiliations:** 1 Department of Pharmacy, The First Affiliated Hospital of Sun Yat-sen University, Guangzhou, China; 2 Department of Infection Prevention and Control, The Affiliated Guangdong Second Provincial General Hospital of Jinan University, Guangzhou, China; 3 Department of Laboratory Medicine, The Affiliated Guangdong Second Provincial General Hospital of Jinan University, Guangzhou, China

**Keywords:** antimicrobial resistance, bloodstream infections, *Enterococcus faecium*, risk factors, vancomycin resistance

## Abstract

**Background:**

Vancomycin-resistant *Enterococcus faecium* (VR-Efm) has shown a rising global trend. This study aimed to analyze the resistance patterns and risk factors of *E. faecium* bloodstream infections (BSIs) in hospitalized patients at a Chinese tertiary hospital in Guangzhou, to provide evidence for infection control.

**Methods:**

This is a retrospective study investigating the antimicrobial susceptibility profiles and clinical characteristics of 48 *E. faecium* isolates recovered from 48 hospitalized patients (one isolate per patient) between 1st January 2022 and 31st December 2024, with all cases meeting the predefined clinical and microbiological diagnostic criteria for bloodstream infections. For patients with multiple positive blood cultures, only the first isolate was included to avoid duplication bias.

**Results:**

A total of 48 patients with BSIs were included in the final analysis. Among these 48 *E. faecium* bloodstream isolates, the vancomycin resistance rate was 31.25% (15/48). The proportion of intensive care unit (ICU) patients was significantly higher in the VR-Efm group than in the vancomycin-susceptible *E. faecium* (VS-Efm) group (73.33% vs. 39.39%, *p* = 0.029). No significant differences were observed between the two groups regarding underlying comorbidities (hypertension, coronary artery disease, and diabetes mellitus), history of drug/food allergies, surgery, hepatitis, tuberculosis, or blood transfusion (*p* > 0.05). Moreover, the mortality rate of patients with VR-Efm BSIs was markedly higher than that of those with VS-Efm infections (46.67% vs. 18.18%, *p* = 0.040). ICU admission (OR = 3.31, 95% CI 1.71–8.48, *p* = 0.029) and prior exposure to glycopeptides (OR = 7.87, 95% CI 1.21–21.16, *p* = 0.031) remained independently associated with VR-Efm infection.

**Conclusion:**

In this single-center retrospective study, the vancomycin resistance rate among *E. faecium* bloodstream isolates was 31.25%. ICU admission and prior glycopeptide exposure were identified as independent risk factors for VR-Efm bloodstream infections. Patients with VR-Efm BSIs had significantly higher mortality than those with VS-Efm infections. Although all isolates remained susceptible to linezolid, the high resistance rate and its association with worse clinical outcomes underscore the urgent need for strengthened antimicrobial stewardship, enhanced surveillance, and targeted infection control measures, particularly in ICUs and among patients with prior glycopeptide exposure.

## Introduction

In recent years, the epidemiology of vancomycin-resistant *Enterococcus faecium* (VR-Efm) has changed markedly, with global surveillance showing a steady rise in vancomycin resistance, particularly among *E. faecium* (1–3). In China, this trend is especially pronounced in Guangdong Province, where studies from 2019 to 2023 have reported a sharp spatiotemporal increase in VR-Efm detection rates from 1.4 to 21.3% (4). This highlights its growing clinical and public health significance. Bloodstream infections caused by VR-Efm pose substantial therapeutic challenges, as treatment options remain largely limited to a few agents, including linezolid, teicoplanin, and daptomycin (5–7).

Despite growing interest in this pathogen, knowledge regarding the clinical impact of VR-Efm infections remains unsatisfactory. Most studies that have focused on the role of vancomycin resistance were carried out before the era of linezolid and under different epidemiological conditions (8). This underscores the critical need for effective clinical management and infection control. Accordingly, the present study aimed to analyze the antimicrobial resistance patterns and associated risk factors of *E. faecium* bloodstream infections in hospitalized patients at a Chinese tertiary care hospital in Guangzhou, providing evidence to guide clinical practice and hospital infection prevention strategies.

## Materials and methods

### Setting

This retrospective study investigated the antimicrobial susceptibility profiles and clinical characteristics of 48 *E. faecium* isolates recovered from 48 hospitalized patients with bloodstream infections between 1 January 2022 and 31 December 2024. All cases met the predefined clinical and microbiological diagnostic criteria for BSIs at the Affiliated Guangdong Second Provincial General Hospital of Jinan University, a tertiary university affiliated hospital in Guangzhou, Guangdong Province, China, with approximately 1,730 beds.

### Study population and inclusion criteria

Patients from whom *E. faecium* isolates were recovered between 1st January 2022 and 31st December 2024 were retrospectively included. Inclusion criteria were age ≥ 18 years and availability of complete medical records, including demographic information, length of hospital stay, ICU admission, underlying medical conditions, clinical outcomes, and other relevant variables. Exclusion criteria were age < 18 years and incomplete clinical data. Patients aged <18 years were excluded because the epidemiology, risk factors, and treatment protocols for *E. faecium* bloodstream infections may differ between pediatric and adult populations, and this study focused specifically on adult hospitalized patients. According to hospitalization outcomes, patients were categorized into a survival (discharged) group and a non-survival (deceased) group for analysis.

### Definition of bloodstream infections

Clinical diagnosis required fever (>38 °C) or hypothermia (<36 °C), with or without chills, plus at least one of the following: Presence of a portal of entry or metastatic focus; Systemic toxic symptoms without an identifiable local infection; Rash, petechiae, hepatosplenomegaly, or neutrophilia with left shift not explained by other causes; Hypotension (systolic BP < 90 mmHg or a drop >40 mmHg from baseline). Microbiological confirmation required either isolation of a pathogen from blood culture or detection of pathogen antigens in blood. For common skin commensals (e.g., coagulase-negative staphylococci), two or more positive cultures from separate occasions were required. For *E. faecium*, a single positive culture was accepted only when accompanied by consistent clinical manifestations and targeted antibiotic therapy; cases lacking clinical correlation were excluded.

### Ethics approval and consent statement

All methods in this study were carried out in accordance with relevant guidelines and regulations. This study was conducted in strict compliance with the Declaration of Helsinki. The study was approved by the Ethics Committee of The Affiliated Guangdong Second Provincial General Hospital of Jinan University, Guangzhou, China (Approval No. 2025-KY-KZ-233-01). Due to the retrospective nature of the study and the use of a de-identified database, the Ethics Committee of The Affiliated Guangdong Second Provincial General Hospital of Jinan University waived the need of obtaining informed consent. No animal studies were conducted in this research. No potentially identifiable images or data are presented in this study.

### Data collection

Specimen Collection and Culture. Blood specimens were collected and inoculated onto appropriate culture media following standard procedures. Bacterial identification and *in vitro* antimicrobial susceptibility testing were conducted using both the disk diffusion method (antibiotic disks from Oxoid, United Kingdom) and antimicrobial susceptibility testing was performed according to the Clinical and Laboratory Standards Institute (CLSI) guidelines (M100, 33rd edition, 2023) using the automated VITEK 2 Compact automated system (bioMérieux, France). The panel of antibiotics tested for *E. faecium* included ampicillin, penicillin, erythromycin, levofloxacin, linezolid, teicoplanin, and vancomycin. Isolates with a vancomycin minimum inhibitory concentration (MIC) of ≥32 μg/mL were classified as resistant.

Demographic and clinical data, including sex, age, length of hospital stay, ICU admission, underlying medical conditions (such as hypertension, coronary artery disease, diabetes mellitus, hepatitis, tuberculosis, drug or food allergies, surgical history, and transfusion history), and outcomes (discharge or death), were extracted from the electronic medical record system (Beijing Jiahe Meikang Information Technology Co., Ltd., version 6.0). In-hospital mortality was defined as death from any cause occurring during the same hospitalization episode in which the *E. faecium* bloodstream infection was diagnosed. Patients were followed until hospital discharge or death, and outcomes were classified as either discharged alive (survival) or deceased (non-survival).

### Statistical analyses

All statistical analyses were conducted using IBM SPSS Statistics software (version 29.0). Categorical variables were compared using the chi-square test and are presented as counts and percentages. The Kolmogorov–Smirnov test was used to assess the normality of continuous variables. For variables not normally distributed, data are presented as medians with interquartile ranges (IQR, P25–P75), and group comparisons were performed using the non-parametric Mann–Whitney *U* test. All statistical analyses were evaluated at the statistical significance level of *p* < 0.05 (two-sided).

## Results

### Antimicrobial susceptibility of *E. faecium* bloodstream isolates

**Figure 1:**
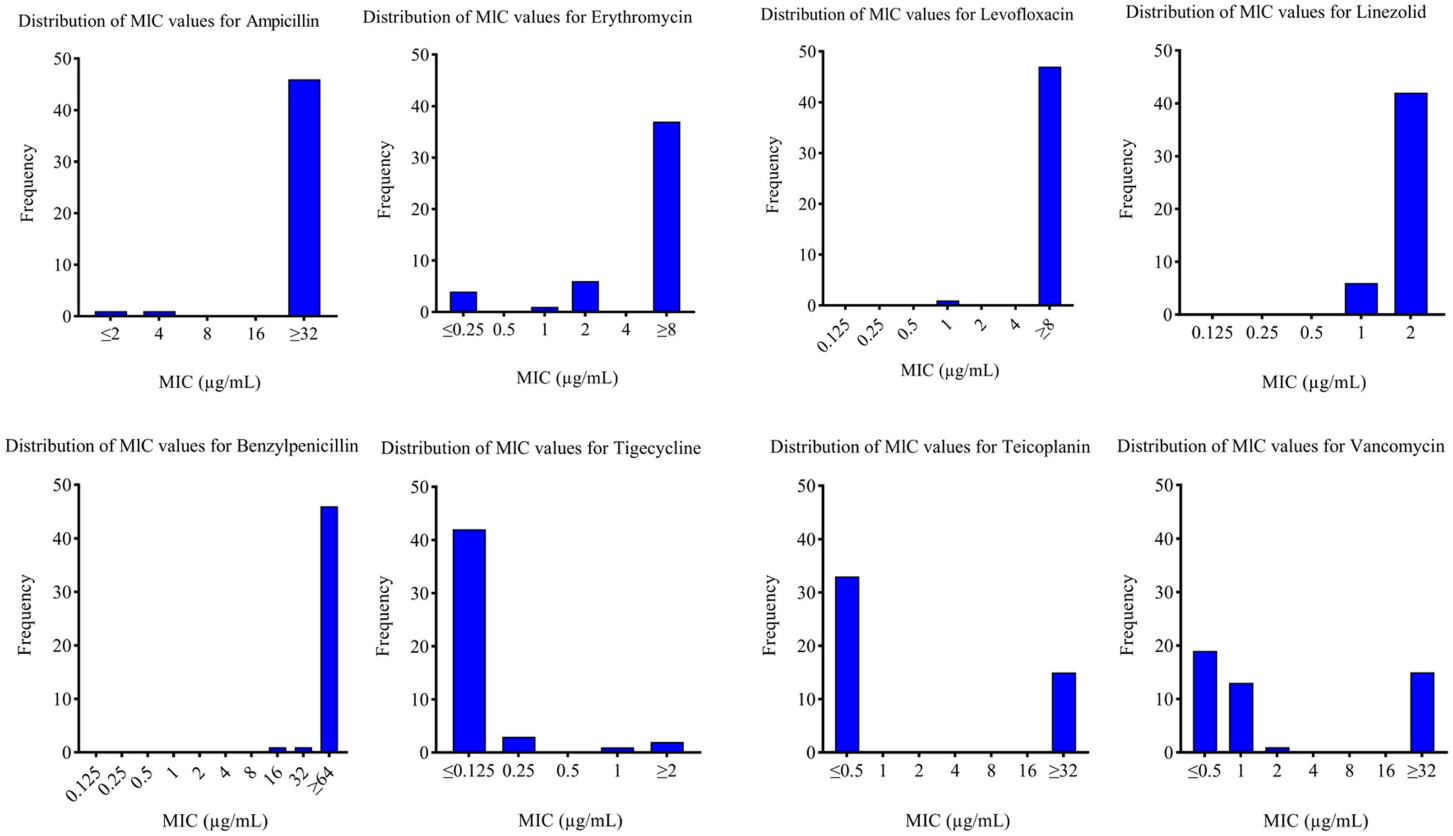
Antimicrobial Susceptibility of *E. faecium*. MIC, minimum inhibitory concentration.

During the study period, 48 *E. faecium* isolates were recovered from blood cultures of hospitalized patients. Antimicrobial susceptibility testing showed high resistance rates to ampicillin (95.83%, 46/48), penicillin (95.83%, 46/48), erythromycin (77.08%, 37/48), and levofloxacin (97.92%, 47/48). Notably, all isolates remained susceptible to linezolid, and 87.50% (42/48) were susceptible to tigecycline. Resistance to vancomycin and teicoplanin was observed in 15 isolates (31.25%) (Figure 1 and Table 1).

**Table 1:** Antimicrobial susceptibility profile of *E. faecium* isolates (*n* = 48).

Antimicrobial agent	MIC breakpoint (μg/mL)	No. of resistant isolates (n)	Proportion (%)
Ampicillin	≥16	46	95.83
Penicillin	≥16	46	95.83
Erythromycin	≥8	37	77.08
Levofloxacin	≥8	47	97.92
Linezolid	≥8	0	0.00
Teicoplanin	≥32	15	31.25
Vancomycin	≥32	15	31.25

MIC, minimum inhibitory concentration. MIC breakpoints are based on CLSI M100 guidelines (33rd edition, 2023).

### Risk factors and clinical outcomes of VR-Efm versus VS-Efm bloodstream infections

Among the 48 patients with *E. faecium* bloodstream infections, 15 were infected with VR-Efm and 33 with vancomycin-susceptible *E. faecium* (VS-Efm). In the VS-Efm group, 60.61% (20/33) were male, compared to 46.67% (7/15) in the VR-Efm group (*p* > 0.05). The median age was 73.0 years (IQR 59.5–82.0) in the VS-Efm group and 79.0 years (IQR 74.0–90.0) in the VR-Efm group, with no statistically significant difference (*p* = 0.078). The median length of hospital stay was 22.0 days (IQR 13.0–39.0) in the VS-Efm group and 18.0 days (IQR 10.0–51.0) in the VR-Efm group (*p* = 0.616). Notably, ICU admission was significantly more frequent among VR-Efm patients (73.33%, 11/15) than in the VS-Efm group (39.39%, 13/33) (*p* = 0.029). No significant differences were observed between the two groups regarding underlying comorbidities (hypertension, coronary artery disease, diabetes mellitus), history of drug/food allergies, surgery, hepatitis, tuberculosis, or blood transfusion (*p* > 0.05). Mortality was significantly higher in the VR-Efm group compared to the VS-Efm group (46.67% vs. 18.18%, *p* = 0.040). The detailed comparison is presented in Table 2. Multivariate logistic regression analysis was performed to identify independent risk factors for VR-Efm bloodstream infections (Table 3). ICU admission (OR = 3.31, 95% CI 1.71–8.48, *p* = 0.029) and prior exposure to glycopeptides (OR = 7.87, 95% CI 1.21–21.16, *p* = 0.031) remained independently associated with VR-Efm infection.

**Table 2:** Comparison of clinical characteristics between patients with VR-Efm and VS-Efm bloodstream infections.

Characteristics	VS-Efm (*n* = 33)	VR-Efm (*n* = 15)	*p*-value
Sex, male	20 (60.61)	7 (46.67)	0.367
Age, years, median [IQR]	73.00 [59.50, 82.00]	79.00 [74.00, 90.00]	0.078
Length of stay, days, median [IQR]	22.00 [13.00, 39.00]	18.00 [10.00, 51.00]	0.616
ICU admission	13 (39.39)	11 (73.33)	0.029
Baseline medical conditions			
Hypertension	15 (45.45)	4 (26.67)	0.217
Coronary heart disease	4 (12.12)	3 (20.00)	0.473
Diabetes mellitus	7 (21.21)	2 (13.33)	0.517
Drug/food allergy history	3 (9.09)	1 (6.67)	0.778
Surgical history	13 (39.39)	6 (40.00)	0.968
Hepatitis	0 (0.00)	2 (13.33)	–
Tuberculosis	0 (0.00)	1 (6.67)	–
Blood transfusion history	4 (12.12)	4 (26.67)	0.210
Invasive procedures			
CVC	18 (54.55)	10 (66.67)	0.430
Urinary Catheter	14 (42.42)	8 (53.33)	0.482
Endotracheal intubation	7 (21.21)	6 (40.00)	0.175
Antimicrobial exposure			
β-lactamase inhibitor	15 (45.45)	6 (40.00)	0.724
Cephalosporins	5 (15.15)	6 (40.00)	0.058
Carbapenems	17 (51.52)	9 (60.00)	0.584
Glycopeptides	2 (6.06)	6 (40.00)	0.003
Quinolones	15 (45.45)	3 (20.00)	0.091
Nitroimidazoles	1 (3.03)	1 (6.67)	0.559
Glycylcyclines	4 (12.12)	0 (0.00)	–
Cephamycins	2 (6.06)	0 (0.00)	–
Oxazolidinones	3 (9.09)	0 (0.00)	–
Polymyxins	3 (9.09)	0 (0.00)	–
Tetracyclines	1 (3.03)	0 (0.00)	–
Infection classification			0.676
Hospital-acquired	5 (15.15)	3 (20.00)	
Community-acquired	28 (84.85)	12 (80.00)	
Outcome			0.040
Death	6 (18.18)	7 (46.67)	
Discharge	27 (81.82)	8 (53.33)	

Data are presented as *n* (%) unless otherwise specified. VR-Efm, vancomycin-resistant *Enterococcus faecium*. VS-Efm, vancomycin-susceptible *Enterococcus faecium*. ICU, Intensive care unit. CVC, Central venous catheter. –, indicates not applicable. Antimicrobial exposure refers to use within 14 days prior to positive blood culture.

**Table 3:** Multivariate analysis of risk factors for VR-Efm bloodstream infections.

Variable	*p* value	OR	95% CI for OR
ICU admission	0.029	3.309	1.707–8.483
Glycopeptides	0.031	7.869	1.210–21.155
Outcome	0.271	0.563	0.107–2.971

ICU, intensive care unit. OR, odds ratio. CI, confidence interval.

## Discussion

Our study demonstrated that the vancomycin resistance rate among *E. faecium* bloodstream isolates was 31.25%. This rate surpasses the 21.3% reported for all isolates by the Guangdong Province multicenter surveillance in 2023 (4). Furthermore, it is significantly higher than the 9.5% prevalence detected among isolates from blood cultures in southern Jiangxi, China in 2024 (9). This distinctive pattern is supported by molecular epidemiological evidence. The ST80 clone has become the predominant circulating strain in Guangdong (4, 10). Its strong environmental adaptability and transmission efficiency may have accelerated the spread of VR-Efm. Moreover, as a commensal member of the human gut microbiota, *E. faecium* possesses the capacity for bidirectional transmission between hospitals and communities. This adds further complexity to its prevention and control.

Several mechanisms may underlie the rapid dissemination of VR-Efm. First, the dominance of the ST80 clone highlights clonal expansion as a key driver. Second, VR-Efm demonstrates remarkable environmental persistence. It can survive on hospital surfaces such as bed rails, monitoring devices, and ventilator circuits. High colonization rates on long-term medical equipment (e.g., dialysis machines, ultrasound probes) further increase transmission risk. Third, cross-contamination via healthcare workers hands and shared equipment amplifies the spread in high risk wards, particularly intensive care units (11, 12).

Consistent with previous studies (13–17), our findings confirmed that VR-Efm bloodstream infections were associated with significantly worse outcomes than vancomycin-susceptible strains, with a mortality rate of 46.67% versus 18.18% (*p* = 0.040). Importantly, patients infected with VR-Efm were more often critically ill, older population, and admitted to the ICU (73.33% vs. 39.39%). Although no linezolid-resistant isolates were detected in our cohort, emerging global reports of linezolid resistance among *Enterococcus* spp. are concerning (18–20). Given its role as one of the last-line therapeutic options, rational use of linezolid must be prioritized to preserve its efficacy.

This study has several limitations. First, it was a single-center retrospective analysis with a relatively small sample size. This may reduce the statistical power of risk factor assessment, limit generalizability, mortality comparison and result in wide confidence intervals in the multivariate analysis. Due to the limited number of VR-Efm cases, the multivariate logistic regression analysis should be interpreted with caution, as the number of events per variable may be insufficient for robust estimation. Wide confidence intervals reflect this limitation. Second, due to incomplete accessibility of antibiotic usage data, we were unable to evaluate the association between vancomycin and linezolid exposure and resistance emergence. Third, molecular characterization of the resistance determinants was not performed. Such data would provide valuable insights into the clonal relatedness and resistance mechanisms of the VR-Efm isolates and should be included in future studies incorporating genomic epidemiology. Future research should adopt a multicenter, prospective design integrating antimicrobial consumption surveillance with molecular epidemiology to better elucidate resistance dynamics and inform targeted infection control strategies.

## Conclusion

In this single-center retrospective study, the vancomycin resistance rate among *E. faecium* bloodstream isolates was 31.25%. ICU admission and prior glycopeptide exposure were identified as independent risk factors for VR-Efm bloodstream infections. Patients with VR-Efm BSIs had significantly higher mortality than those with VS-Efm infections. Although all isolates remained susceptible to linezolid, the high resistance rate and its association with worse clinical outcomes underscore the urgent need for strengthened antimicrobial stewardship, enhanced surveillance, and targeted infection control measures, particularly in ICUs and among patients with prior glycopeptide exposure.

## Data Availability

The original contributions presented in the study are included in the article/supplementary material, further inquiries can be directed to the corresponding author.
